# Functional Status Predicts Acute Care Readmissions from Inpatient Rehabilitation in the Stroke Population

**DOI:** 10.1371/journal.pone.0142180

**Published:** 2015-11-23

**Authors:** Chloe Slocum, Paul Gerrard, Randie Black-Schaffer, Richard Goldstein, Aneesh Singhal, Margaret A. DiVita, Colleen M. Ryan, Jacqueline Mix, Maulik Purohit, Paulette Niewczyk, Lewis Kazis, Ross Zafonte, Jeffrey C. Schneider

**Affiliations:** 1 Department of Physical Medicine and Rehabilitation, Spaulding Rehabilitation Hospital, Harvard Medical School, Boston, Massachusetts, United States of America; 2 Department of Physical Medicine and Rehabilitation, Massachusetts General Hospital, Harvard Medical School, Boston, Massachusetts, United States of America; 3 Department of Neurology, Massachusetts General Hospital, Harvard Medical School, Boston, Massachusetts, United States of America; 4 Uniform Data System for Medical Rehabilitation, Amherst, New York, United States of America; 5 Daemen College, Health Care Studies Dept., Amherst, New York, United States of America; 6 Sumner Redstone Burn Center, Surgical Services, Massachusetts General Hospital, Harvard Medical School, Boston, Massachusetts, United States of America; 7 Shriners Hospitals for Children®-Boston, Boston, Massachusetts, United States of America; 8 Neurorehabilitation and Traumatic Brain Injury, National Intrepid Center of Excellence: Intrepid Spirit One, Fort Belvoir Community Hospital, Fort Belvoir, Virginia, United States of America; 9 Department of Health Policy and Management, Boston University School of Public Health, Boston, Massachusetts, United States of America; University of Pennsylvania, UNITED STATES

## Abstract

**Objective:**

Acute care readmission risk is an increasingly recognized problem that has garnered significant attention, yet the reasons for acute care readmission in the inpatient rehabilitation population are complex and likely multifactorial. Information on both medical comorbidities and functional status is routinely collected for stroke patients participating in inpatient rehabilitation. We sought to determine whether functional status is a more robust predictor of acute care readmissions in the inpatient rehabilitation stroke population compared with medical comorbidities using a large, administrative data set.

**Methods:**

A retrospective analysis of data from the Uniform Data System for Medical Rehabilitation from the years 2002 to 2011 was performed examining stroke patients admitted to inpatient rehabilitation facilities. A Basic Model for predicting acute care readmission risk based on age and functional status was compared with models incorporating functional status and medical comorbidities (Basic-Plus) or models including age and medical comorbidities alone (Age-Comorbidity). C-statistics were compared to evaluate model performance.

**Findings:**

There were a total of 803,124 patients: 88,187 (11%) patients were transferred back to an acute hospital: 22,247 (2.8%) within 3 days, 43,481 (5.4%) within 7 days, and 85,431 (10.6%) within 30 days. The C-statistics for the Basic Model were 0.701, 0.672, and 0.682 at days 3, 7, and 30 respectively. As compared to the Basic Model, the best-performing Basic-Plus model was the Basic+Elixhauser model with C-statistics differences of +0.011, +0.011, and + 0.012, and the best-performing Age-Comorbidity model was the Age+Elixhauser model with C-statistic differences of -0.124, -0.098, and -0.098 at days 3, 7, and 30 respectively.

**Conclusions:**

Readmission models for the inpatient rehabilitation stroke population based on functional status and age showed better predictive ability than models based on medical comorbidities.

## Introduction

Nearly one fifth of Medicare patients discharged from a hospital are rehospitalized within 30 days due to an acute medical condition [1]. Recently discharged patients have been postulated to have increased vulnerability to subsequent medical complications due to a ‘post-hospital syndrome’ resulting from additive effects of their original medical illness and the stresses of hospitalization itself [1]. Fiscal and regulatory changes implemented by the Center for Medicare and Medicaid Services (CMS) aimed at reducing readmissions within 30 days of acute care discharge have prompted an increasing number of studies that attempt to identify the causes of acute care readmissions and develop predictive models of rehospitalization with the aim of developing preventive strategies and interventions [2].

Relatively stable rates of reported all-cause readmissions, despite the identification of a growing number of possible risk factors, suggest that the reasons for acute care readmissions are complex and multifactorial [3]. While various risk prediction models have examined patient- and system-level factors at different time points as potential contributors to acute care readmissions, most risk prediction models tested in larger populations have demonstrated poor discriminative ability [2]. Models using readily available, retrospective administrative data have intuitive appeal given their potential application for hospital comparison purposes and use for developing standardized intervention strategies that may be customized to particular institutional settings. Many published risk prediction models that have been developed for acute care readmissions have included the presence of comorbidities and demonstrate modest predictive ability [2–4]. In a recent risk prediction model for 30-day potentially avoidable readmissions, Donzé and colleagues suggested that markers of illness severity or clinical instability may improve model performance beyond the presence of medical comorbidities alone [5]. By comparison, risk prediction models infrequently account for functional status as a contributing variable, despite hypothesized improvements in models’ predictive ability [2]. While specific and quantifiable information regarding functional status may be difficult to obtain from retrospective acute care datatsets, standardized measurements of functional status and the presence of medical cormorbidities are routinely collected for the inpatient rehabilitation population.

Several studies in relatively small populations found a significant relationship between functional status and acute care readmission from the post-acute inpatient rehabilitation setting, although these did not examine 30-day readmission risk specifically [6–9]. Risk prediction models developed for the inpatient rehabilitation burn injury population using a large, retrospective administrative data set showed functional status to be an important predictive variable. Notably, the addition of medical comorbidities to models using functional status did not demonstrate enhanced predictive ability [10,11].

This study examines the role of functional status compared to that of medical comorbidities in risk prediction models for acute care readmissions in the inpatient rehabilitation stroke population at different time points following acute care discharge.

## Methods

This study uses a retrospective cross-sectional study design. We hypothesized that functional status would predict acute care readmissions and that its inclusion in risk prediction models would yield better predictive ability than models based on the presence of comorbidities alone. We analyzed data from the Uniform Data System for Medical Rehabilitation (UDSMR), a repository for inpatient rehabilitation facility (IRF) functional outcome data. CMS requires IRFs to complete the Inpatient Rehabilitation Facility Patient Assessment Instrument (IRF-PAI), which contains demographic, social, medical, and functional data. UDSMR serves approximately 70% of all IRFs in the United States (data in S1 Text). Data were obtained from the UDSMR from 2002–2011. Inclusion criteria were Medicare-established Impairment Group Codes for IRF admission of 01.1–01.9 (indicating right, left, bilateral body involvements in stroke; no paresis; or other stroke) [12]. Exclusion criteria were age greater than 108 years, length of stay at acute care facility greater than 90 days (onset days > 90), admission to IRF from a facility other than an acute care hospital or from home, and death in the IRF setting. This study received exemption from the Institutional Review Board at Spaulding Rehabilitation Hospital given the de-identified nature of the data set.

Information collected in the IRF-PAI includes up to 10 comorbidities, coded according to the International Classification of Diseases 9th edition Clinical Modification (ICD-9-CM). Transfer to an acute care hospital is designated as a disposition category within the IRF-PAI. The IRF-PAI also includes the FIM™ instrument, a standardized evaluation tool that assesses function and serves as a proxy for the intensity of resources required for care [13–15]. The FIM™ instrument consists of eighteen items in either a motor or cognitive domain, each of which is rated on a seven-level ordinal scale from completely dependent (1) to independent (7) [16].The motor domain consists of 13 items, which include eating, dressing, grooming, bathing, toileting, sphincter control, bowel and bladder management, transfers, and locomotion. The cognitive domain consists of 5 items regarding comprehension, expression, problem-solving, social interaction and memory (S1 Table). IRF-PAI data were analyzed using Stata version 12.0 and presented in accordance with the STROBE guidelines [17].

Medical comorbidities, designated by ICD-9-CM code in the IRF-PAI, were further analyzed according to three different comorbidity scoring systems: the Elixhauser comorbidity method (Healthcare Cost and Utilization Project, Comorbidity Software v.3.7, 2012; Office of Communications and Knowledge Transfer, Agency for Healthcare Research and Quality, Rockville MD) [18,19], Deyo adaptation of the Charlson Comorbidity Index [20,21], and the Centers for Medicare & Medicaid Services (CMS) Comorbidity Tiers (S2 Table). Logistic regression analysis was used to create models in which the odds of transfer to an acute care hospital was the dependent variable and functional status at admission, medical comorbidities, and age were independent variables. We examined acute care readmissions for the inpatient rehabilitation stroke population at three separate time points: 3, 7, and 30 days following acute care discharge.

First, we created a pre-specified ‘Basic Model’ that included 3 variables related to age and functional status: age, FIM™ motor score, and FIM™ cognitive score. Next, we compared the performance of the Basic Model to models that added comorbidity data to the Basic Model according to each of the three comorbidity scoring systems (Basic-Plus models), and models that included only age and comorbidities from each scoring system (Age-Comorbidity models). Consequently, three Basic-Plus models and three Age-Comorbidity models were developed (Table 1). For each model, we investigated predictive ability at 3 days, 7 days, and 30 days into the rehabilitation stay. We hypothesized that the Basic Model would perform similarly to the Basic-Plus models and demonstrate improved predictive ability compared to the Age-Comorbidity models at all three time points.

**Table 1:** Logistic regression models.

**Basic Functional Model**	Age, FIM motor score, FIM cognitive score
**Basic Plus Elixhauser**	Age, FIM motor score, FIM cognitive score, Elixhauser comorbidities
**Basic Plus Deyo**	Age, FIM motor score, FIM cognitive score, Deyo-Charlson Comorbidity Index sum scores*
**Basic Plus CMS Tiers**	Age, FIM motor score, FIM cognitive score, CMS Comorbidity Tiers classification
**Age + Elixhauser**	Age, Elixhauser comorbidities
**Age + Deyo**	Age, 2 Deyo-Charlson Comorbidity Index sum scores*
**Age + CMS Tiers**	Age, CMS Comorbidity Tiers classification

*Deyo-Charlson sum scores are calculated as follows: The first sum score is based on summing the total number of comorbidities that a subject has that are on the Deyo-Charlson index. The second sum score is the total number of points from the Charlson index that the patient has.

The area under the receiver operator curve (C-statistic) was used to test model performance. The C-statistic is defined as the proportion of time a model correctly discriminates between a pair of high- and low-risk individuals and has been used to describe discriminative ability of models in prior readmission studies and in a systematic review of readmission risk prediction models [2]. A C-statistic of 0.50 signifies that a model performs no better than chance, a C-statistic of 0.70 to 0.80 signifies modest or acceptable discriminative ability, and a C-statistic of greater than 0.80 signifies good discriminative ability [22,23]. We used the difference between C-statistics for two models at the same time point as a comparison method. A C-statistic difference of 0.05 in model comparisons was selected as meaningful based on prior literature [24]. Any Basic Plus model meeting this C-statistic threshold and any failure of the Basic Model to outperform an Age-Comorbidity model by at least +0.05 would be considered evidence against our hypothesis. Tests of significance were not performed on the differences between C-statistics calculated from our models as this is not routinely performed on the differences between C-statistics for models using large administrative data sets, since even negligible differences are probabilistically expected to be statistically significant given large sample sizes such as our own [25,26]. Cross-validation was performed using a k-fold random cross-validation procedure with 10 splits to verify that regression weights were not sample-dependent. Model calibration curves were assessed at 3, 7, and 30 days based on C-statistic criteria in order to assess the model’s ability to distinguish individuals in various risk categories at different time points.

## Results

### Patient Characteristics

The UDSMR database of adult IRF discharges comprised 4,467,307 total cases between 2002 and 2011. We excluded 19,177 patients who left the IRF setting against medical advice, leaving 4,448,130 eligible cases. Of these, 901,652 had an Impairment Group code of stroke. We excluded 26 patients with ages recorded as greater than 108 as presumed documentation errors, 30,135 patients with a delay of >90 days between stroke onset and IRF admission, 38,799 patients who were not admitted to inpatient rehabilitation directly from an acute hospital, and 1,865 patients who died while admitted to inpatient rehabilitation. The final sample size was 803,124 patients from 1,157 IRFs (S1 Fig). Of these, 88,187 (11%) patients were transferred back to an acute hospital including 22,247 (2.8%) transferred within 3 days, 43,481 (5.4%) transferred within 7 days, and 85,431 (10.6%) transferred within 30 days after IRF admission. Table 2 shows demographic, medical and facility data for the study population.

**Table 2:** Patient Characteristics.

**Number of patients, *n***	803,124
**Number of facilities, *n***	1157
**Age, mean (SD)**	69.78 (13.78)
**Male, *n* (%)**	388,235 (48.35)
**Race, *n* (%)**	
Caucasian	578,240 (72.00)
African American	127,120 (15.83)
Latino/Hispanic	47,483 (5.91)
Asian	22,099 (2.75)
American Indian / Alaskan	3,547 (0.44)
Hawaiian / Pacific Islander	4,438 (0.55)
Multiracial	2,552 (0.32)
Missing	17,645 (2.20)
**Married, *n* (%)**	393,857 (49.04)
**Living alone, *n* (%)**	216,866 (27.0)
**Employed pre-injury, *n* (%)**	133,832 (16.66)
**Primary payer source, *n* (%)**	
Medicare	554,897 (69.09)
Medicaid	46,328 (5.77)
Workers Compensation	464 (0.06)
Unreimbursed	6,366 (0.79)
Commercial	66,256 (8.25)
Other	128,813 (16.04)
**Number of comorbidities, mean (SD)**	7.8 (2.59)
**Onset days, mean (SD)**	9.06 (9.73)
**Length of IRF stay, mean days (SD)**	16.63 (10.19)
**Operating beds, mean (SD)**	45.24 (36.39)
**FIM Admission rating, mean(SD)**	55.96 (19.71)
**FIM Discharge rating, mean (SD)**	80.56 (24.39)
**Discharge disposition, *n* (%)**	
Community	556,166 (69.26)
Acute facility	88,187 (10.98)
Skilled nursing/subacute	100,207 (12.49)
Other	59,640 (7.43)

### Regression Model Results

Logistic regression coefficients for the Basic Model at each time point are shown in Table 3. There were minor (approximately 0.01) differences in coefficients across the 10 internal cross-validation models, suggesting that the regression coefficients are not sample-dependent. Table 4 shows the C-statistic for each model at each time point. The C-statistics for the Basic Model are 0.701, 0.672, and 0.682 at days 3, 7, and 30 respectively. The Basic-Plus Model C-statistics were marginally better at each time point, though not by the threshold of 0.05. The Basic Model performed substantially better than the three Age-Comorbidity models at each time point. As compared to the Basic Model, the best-performing Basic-Plus model was the Basic+Elixhauser model with C-statistics differences of +0.011, +0.011, and + 0.012, and the best-performing Age-Comorbidity model was the Age+Elixhauser model with C-statistic differences of -0.124, -0.098, and -0.098 at days 3, 7, and 30 respectively. Model calibration for the Basic Model at each time point was good based on the calibration curves (S2 Fig).

**Table 3:** Logistic Regression Coefficients for the Basic Model.

	3 days	7 days	30 days
Age	1.004 (1.003, 1.005)	1.005 (1.004, 1.006)	1.004 (1.003,1.005)
FIM motor	0.954 (0.952, 0.955)	0.963 (0.962,0.964)	0.958 (0.957,0.959)
FIM cognitive	0.979 (0.976, 0.982)	0.980 (0.978, 0.982)	0.984 (0.983,0.986)
Constant	0.137 (0.121, 0.155)	0.203 (0.183, 0.224)	0.478 (0.442,0.517)

Data presented as Coefficient (95% Confidence Interval).

**Table 4:** C-statistics (see Table 2 for model descriptions).

	Basic Model	Basic Plus models			Age Comorbidity models		
	Age + FIM	Basic + Elixhauser	Basic + Deyo	Basic + CMS Tiers	Age + Elixhauser	Age + Deyo	Age + CMS Tiers
**3 days**	0.701	0.712	0.702	0.703	0.577	0.540	0.544
**7 days**	0.672	0.683	0.674	0.673	0.574	0.545	0.552
**30 days**	0.682	0.694	0.685	0.687	0.584	0.553	0.575

## Discussion

While functional status has been suggested as an important predictor of acute care readmissions, it remains comparatively understudied in studies of larger post-acute populations in the United States and larger, population-based or multi-center studies of readmission risk have generally demonstrated modest discriminative ability (C-statistic range 0.55–0.65) [2]. Several smaller studies of the inpatient rehabilitation stroke population have demonstrated the ability of functional status to predict the risk of readmissions or transfer to an acute facility but have not examined the risk of 30-day readmissions specifically [6–9]. In contrast to many acute care administrative data sets, standardized information on functional status is readily available in large data repositories for the inpatient rehabilitation setting, since it is collected consistently using a set evaluation tool (FIM™ instrument) for the purposes of estimating expected rehabilitative care needs and resource allocation. The present study builds on contemporary research underlining functional status as an important predictor of acute care readmissions and demonstrates the feasibility of incorporating a basic measure of functional status (FIM™ instrument) into risk prediction models for the stroke population undergoing inpatient rehabilitation by using existing administrative data sets.

This study is the first to develop readmissions models using data obtained from a large, national data set and showed that age and functional status on admission predict the risk of acute care readmissions well and with good model calibration at 3, 7, and 30 days from IRF admission. Models incorporating age and functional status (Basic Models) on admission alone consistently outperformed models based on age and comorbidities (Age-Comorbidity Models). The addition of comorbidities to these basic models (Basic Plus Models) increased complexity but did not significantly enhance predictive ability at 3, 7, or 30 days. Risk prediction for the Basic Model at 3 days in particular showed substantially better predictive ability compared with large, population-based models incorporating comorbidities in prior literature [2], while risk prediction of the Basic Model at 7 and 30 days was slightly less. Overall models’ predictive ability at 3, 7, or 30 days did not differ significantly.

There are several potential reasons for the improved predictive ability of models incorporating functional status as compared with models that rely on medical comorbidities for readmissions in the inpatient rehabilitation stroke population. While ICD-9-CM codes indicating the presence of medical comorbidities may be easily obtained from large, administrative data sets, information regarding disease severity and clinical instability is typically sparse and has been proposed as a key to improving models’ predictive ability [5]. By contrast, functional status is expressed as a basic level of impairment using admission FIM™ instrument scores in this study and as such, may be a more accurate representation of illness severity by proxy than individual comorbidity codes. Alternatively, poor functional status may contribute to readmission risk through an increased incidence of complications due to immobility. For example, one study showed that urinary tract infections, chest infections, percutaneous endoscopic gastrostomy complications, and falls—complications typically associated with poor mobility—were responsible for a large fraction of readmissions in stroke patients [25].

Interventions aimed at promoting successful recovery after hospitalization and reducing acute care readmissions often focus on improving care for specific medical conditions (e.g. care performance measures for pneumonia, heart failure, and acute myocardial infarction in the acute care setting) and transitions of care at nodal time points (e.g. direct communication between inpatient and outpatient providers prior to acute care discharge) [1,3]. The results of this study showed that functional status remained a superior predictor of readmissions at 3, 7, and 30 days compared with comorbidities alone, despite potential differences in immediate or short-term risk following acute care discharge [26]. These findings suggest that basic interventions to help maintain or improve functional status and mobility during hospitalization may help mitigate a potentially modifiable risk factor for acute care readmissions in stroke patients discharged to inpatient rehabilitation. A growing body of research on the mobilization of patients in the intensive care unit (ICU) setting may inform efforts to prevent or alleviate physical deconditioning during acute hospitalization, especially for patients with critical illness [27]. Hoyer and colleagues recently observed an association between reduced functional status and increased acute care readmission risk across patient populations admitted to an IRF with neurologic, orthopedic, and medical categories, implicating that an emphasis on managing functional impairments during acute hospitalization may lead to improved clinical outcomes more generally [28].

The results of this study must be interpreted within the context of their limitations. This was an observational study and we are unable to demonstrate a direct, causative relationship between functional status and readmissions using our data set. Comorbidity data was obtained from ICD-9-CM codes, of which a maximum of 10 per patient are recorded, rather than all potential comorbidities. Furthermore, the documented presence of medical comorbidities in isolation is not a reliable indication of illness severity or clinical stability. We addressed this limitation by using three different and validated comorbidity scoring systems in the development of our risk prediction models to account for medical comorbidities using a standardized measure. Demographic and social information is collected within the IRF-PAI but was not included in our models because of the hypothesis-driven design of our study. However, prior studies have suggested that the relationship between functional status and readmissions in the inpatient rehabilitation stroke population are minimally confounded by demographic factors [6,7,9]. While stroke symptomatology and measurements of acute severity such as the National Institutes of Health Stroke Scale (NIHSS) have been shown to predict disposition and certain functional outcomes in neurologic recovery, this study examines functional status measured with the FIM™ instrument, a more general, global measure of function. This is the first large study using a national database to examine the relationship between functional status and readmission risk and a limitation of our data set is a lack of information on acute stroke symptomatology and presentation. The FIM™ instrument was designed for use in the IRF setting, and is typically administered by nursing staff and therapists who have completed a basic level of specialized training. Despite the resources required to administer, the FIM™ instrument retains its appeal in the IRF setting due to consistent data collection and documentation practices. However, this may create challenges for implementing its use at other levels of care. Future research may help to determine whether specific measures of function and mobility such as the Barthel Index and the modified Rankin Scale have similar power to predict risk of readmission from other levels of care in conjunction with age, or whether newer, abbreviated measures of function designed for use during acute hospitalization such as the AlphaFIM™ may prove useful in the acute care setting [29,30]. Due to the retrospective, cross-sectional study design these findings require prospective validation, for which generating large sample sizes may be difficult. The models in this study were validated using k-folds cross-validation to improve overall generalizability.

## Conclusions

Functional status effectively predicts acute care readmissions after stroke in the inpatient rehabilitation setting. Models using functional status as indicated by FIM™ instrument scoring and age showed improved predictive ability compared with medical comorbidities and age alone in the inpatient rehabilitation stroke population using data from a large, administrative data set. Our data adds to increasing evidence that functional status is an important measure of health and predictor of risk for adverse health events following acute hospitalization in the stroke population [7,31–36]. Moreover, these results suggest that efficient and systematic clinical assessment of function and strategies to maintain functional status and reduce immobility during acute hospitalizations may prove to be essential components of effective interventions to reduce acute care readmissions. The identification of key elements to create high-quality, cost-effective strategies to reduce and prevent acute care readmissions is an area of future inquiry that has critical significance.

## Supporting Information

S1 Fig Flow Diagram. (PDF)

S2 Fig Model calibration curves and comparison. (DOC)

S1 Table FIM™ Instrument Components. (DOC)

S2 Table Comorbidity scoring and analysis methods. (DOC)

S1 Text USDMR Data Set Components. (DOC)
