# Supplementary figures and images for: Functional Status Predicts Acute Care Readmissions from Inpatient Rehabilitation in the Stroke Population
Source: PLoS One. 2015 Nov 23;10(11):e0142180. doi: 10.1371/journal.pone.0142180 (PMC4657881; doi:10.1371/journal.pone.0142180)

**S1 Figure. Flow Diagram.**

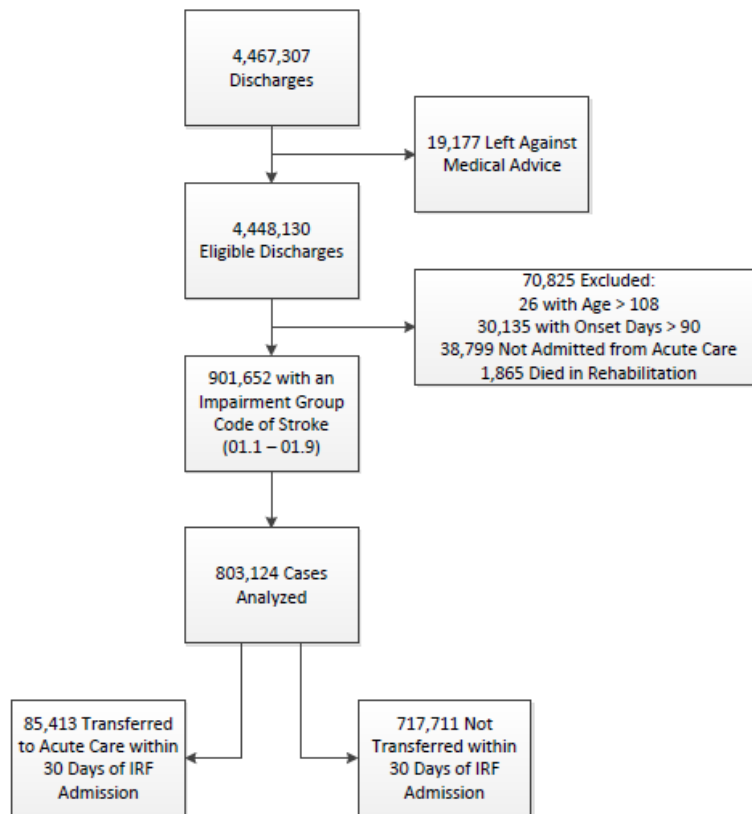

Supplement: S1 Fig — (PDF) [file pone.0142180.s001.pdf]
