# Supplementary material for: Functional Status Predicts Acute Care Readmissions from Inpatient Rehabilitation in the Stroke Population
Source: PLoS One. 2015 Nov 23;10(11):e0142180. doi: 10.1371/journal.pone.0142180 (PMC4657881; doi:10.1371/journal.pone.0142180)
Supplement: S2 Fig — (DOC) [file pone.0142180.s002.doc]

**S2 Figure. Model calibration curves and comparison.**


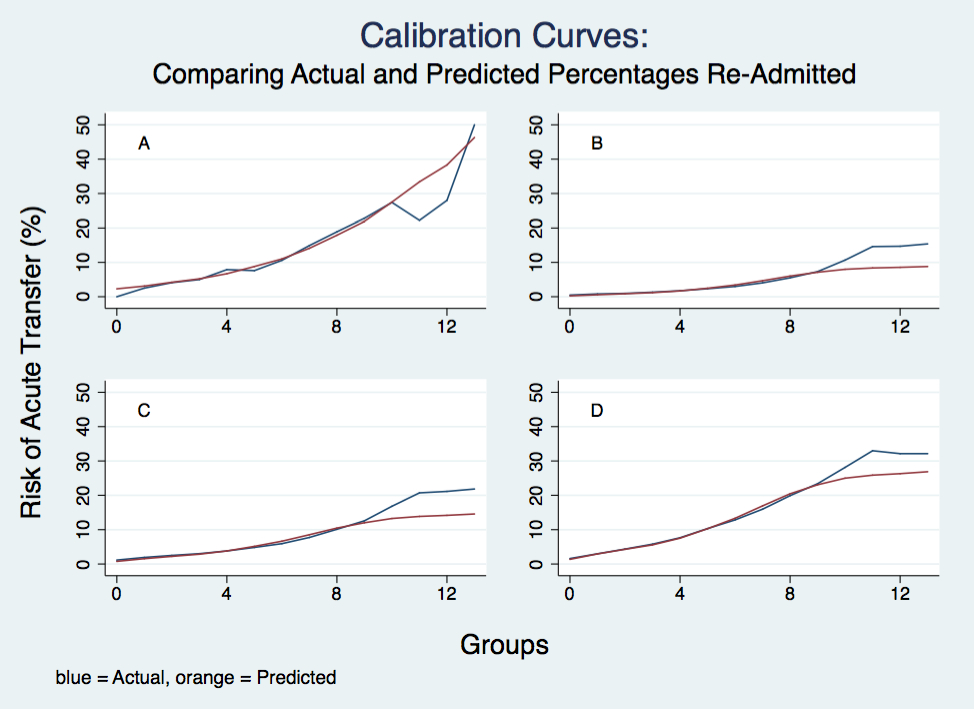


Calibration curves demonstrating actual versus predicted readmission rates for risk groups. Our model is compared with a prior readmissions risk prediction model with excellent fit developed at a single institution, shown in panel A.[^1^](#_ENREF_1) Panels B, C, and D, display results for the Basic models derived in this study within the first 3, 7, and 30 days respectively.

1. Donze J, Aujesky D, Williams D, Schnipper JL. Potentially avoidable 30-day hospital readmissions in medical patients: derivation and validation of a prediction model. JAMA Intern Med 2013;173(8):632-8.
