# Supplementary material for: Functional Status Predicts Acute Care Readmissions from Inpatient Rehabilitation in the Stroke Population
Source: PLoS One. 2015 Nov 23;10(11):e0142180. doi: 10.1371/journal.pone.0142180 (PMC4657881; doi:10.1371/journal.pone.0142180)
Supplement: S2 Table — (DOC) [file pone.0142180.s004.doc]

**S2 Table. Comorbidity scoring and analysis methods.**

Summary of the comorbidity measurements used in the analysis.

| Index | Descrition |
| --- | --- |
| Elixhauser comorbidity method | The Elixhauser comorbidity scoring method is comprised of 29 disease categories consisting of specific ICD-9-CM codes corresponding with comorbidities in each category. |
| Deyo-Charlson Comorbidity Index | The Charlson Comoribidty Index is another comorbidity classification system. It combines multiple comorbidities into a weighted cumulative score which was adapted for use with ICD-9-CM codes (Deyo-Charlson Index). |
| CMS Comorbidity Tiers | The CMS Comorbidity Tiers utilizes a four-tiered system for grading medical complexity as part of its prospective payment system assigned grades A-D. Those assigned to comorbidity tier A have no additional cost, while those assigned to tiers B, C, and D have high, medium and low predicted costs respectively. |
