# Supplementary material for: Functional Status Predicts Acute Care Readmissions from Inpatient Rehabilitation in the Stroke Population
Source: PLoS One. 2015 Nov 23;10(11):e0142180. doi: 10.1371/journal.pone.0142180 (PMC4657881; doi:10.1371/journal.pone.0142180)
Supplement: S1 Text — (DOC) [file pone.0142180.s005.doc]

**S1 Text. USDMR Data Set Components.**

The Uniform Data System for Medical Rehabilitation, a division of UB Foundation Activities, Inc. is a repository for inpatient rehabilitation facility (IRF) functional outcome data. The Centers for Medicare and Medicaid Services (CMS) require IRFs to complete the Inpatient Rehabilitation Facility Patient Assessment Instrument (IRF-PAI), which contains demographic, social, medical, and functional data as measured by FIM™ instrument rating. UDSMR serves approximately 70% of all IRFs in the United States.

Facilities are assigned de-identified codes by UDSMR to protect the identity of the subscribing facilities. Demographic data include age, gender, race, marital status, occupational status pre-injury, pre-hospital living situation (alone or with others) and primary payer source (Medicare, Medicaid, workers compensation, unreimbursed, commercial, other). Medical data include length of time from injury to IRF admission (onset days), pre-rehabilitation setting (home, acute facility, other), IRF admission day of week, whether or not this was the first rehabilitation admission for the current diagnosis, and IRF length of stay and medical comorbidities. Functional data available are admission and discharge FIM™ ratings, as well as deficits in swallowing. Facility data include operating beds, Commission on the Accreditation of Rehabilitation Facilities (CARF) certification and facility patient volume.
